# Alterations in Mycelial Morphology and Flow Cytometry Assessment of Membrane Integrity of *Ganoderma boninense* Stressed by Phenolic Compounds

**DOI:** 10.3390/biology10090930

**Published:** 2021-09-18

**Authors:** Daarshini Ganapathy, Yasmeen Siddiqui, Khairulmazmi Ahmad, Fariz Adzmi, Kong Lih Ling

**Affiliations:** 1 Laboratory of Sustainable Agronomy and Crop Protection, Institute of Plantation Studies, Universiti Putra Malaysia, Serdang 43400, Malaysia; daarshinig@gmail.com (D.G.); farizadzmi@upm.edu.my (F.A.); lihling@upm.edu.my (K.L.L.); 2 Department of Plant Protection, Faculty of Agriculture, Universiti Putra Malaysia, Serdang 43400, Malaysia

**Keywords:** oil palm, basal stem rot, phenolic compounds, ergosterol, membrane disruption, flow cytometer, membrane permeability

## Abstract

**Simple Summary:**

Oil palm is an important cash crop in the tropics, producing 3–8 times more oil than any other oil crop. Basal stem rot (BSR) disease caused by *Ganoderma* spp is one of the greatest threats to oil palm producing countries, leading to economic losses. The pathogen infection has a lethal effect on oil palm by degrading the xylem and tissues of the palm bole, which subsequently restricts water and nutrient uptake from the roots to the upper parts of the palm, leading to stress and making it vulnerable to further saprophytic microbial attack. The progression of the disease is very slow; however, every infected plant eventually dies and, hence, *Ganoderma* is known as the ‘silent killer’ of oil palm. There are lots of strategies that have been tested and carried out by the researchers to overcome the losses caused by BSR; however, little success has been achieved. This study assessed naturally occurring phenolic compounds as a green control measure to suppress the pathogen and identify their effect on the cell membrane potential as a possible mechanism of suppression. The utilization of phenolic compounds may help in reducing the usage of synthetic fungicides and produce less impact on the environment.

**Abstract:**

Global increase in demand for palm oil has caused an intensification in oil palm plantation; however, production is greatly hindered by Basal Stem Rot (BSR) disease caused by *Ganoderma boninense*. There are many approaches to controlling BSR, although, there is no accurate, sustainable and effective method to suppress *G. boninense* completely. Hence, four phenolic compounds [Gallic acid (GA), Thymol (THY), Propolis (PRO) and Carvacrol (CARV)] were selected to evaluate their antifungal effect, ability to alter the mycelium morphology, and fungal cell integrity against *G. boninense*. Significant differences (*p* < 0.05) were observed and 94% of inhibition was exerted by GA on *G. boninense* growth. Scanning Electron Microscopy and High-Resolution Transmission Electron Microscopy observations revealed that GA and THY treatment caused severe damage to the mycelium and recorded the highest amount of sugar and electrolyte leakage. The study of cell integrity and morphological disruption has elucidated the reduction of *G. boninense* cell viability. Generally, our findings confirm the fungistatic effects of GA and THY. The evolution of phenolic compounds during the phytopathology studies indicated their coherence in eradicating the *G. boninense*. It is proposed that GA and THY had the potential to be developed further as a natural antifungal treatment to suppress *G. boninense*.

## 1. Introduction

Malaysia has one of the biggest oil palm industries, which contributes about 9.5% and 19.7% of the world’s palm oil production and exports respectively, with 85% of total palm oil coming from Malaysia and Indonesia [1]. Economic development and its coexistent productivity gains enable the increase in demand for the oil palm’s (*Elaeis guineensis* Jacq.) industrial value [2]. For example, the extended shelf-life of oil, boosting nutritional and health contents have caused the oil palm to become a major crop in the world, especially in South-East Asian countries [3]. Moreover, the palm produces 3–8 times more oil than any other oil crop, including canola, sunflower, soy and rapeseed [4]. High output, easy establishment, and low cost of production system make the crop very profitable [3,5,6]. Indeed, oil palm is a commercial and economically valued cash crop that benefits Malaysia, assisting to further diversify its economy. It contributes commercially as one of the major turning points of the plantation and export industry [7]. As reforestation and forest conversion into the oil palm sector became the dominant pattern in advanced economies, relative reliability of its supply and agricultural productivity improved drastically and has ensured until now [8]. Additionally, it has been documented that there has been a five-fold increase in the production of oil palm over 10 years [9]. Consequently, we can deduce that a drastic need for sustainable production of oil palm has been a major concern, globally [10,11].

The existing oil palm cultivated area is consistently prone to attack by several diseases [12] and fruit-bunch yield reduction caused by pests and diseases may contribute up to a yield loss of ~50 to 80% [13]. Basal stem rot (BSR) caused by the fungus *Ganoderma boninense*, the most aggressive representative compared to other *Ganoderma* spp, is of major concern for sustainability in Malaysia and Indonesia and it is predicted that more than 60 million mature oil palm could be infected in Malaysia [14,15]. This pathogen caused severe damage to the oil palm plantations, a crisis in the production of the oil palm sector, which subsequently led to economic losses estimated at $365 million per annum [16,17]. The symptoms of BSR are hardly visible at the early stage and by the time it is recognized, it is too late to eradicate the pathogen infection and disease expansion is out of control. Young palms usually die within 6–24 months after the invasion of pathogen, whereas mature palms takes about another 2–3 years after the emergence of first symptoms [11]. The BSR disease infection destroys the structure and lignin within the tree [15], extensively disrupting the stem and root system [11]. The roots are usually found to be completely colonized and dead before the emergence of foliar symptoms. Therefore, failure to recognize the progression of BSR has led into complications in current cultivation and for the next generation of planting [11].

Many strategies have been tested and carried out by the researchers to overcome the losses caused by BSR by improving the experimental researches from modern breeding strategies to more sophisticated production technologies [18]. The biological control strategies have been imposed, having some good impacts on disease suppression but generally not tested with in-field evaluation [11]. Moreover, studies have proved that chemical agents help in the reduction of white-rot fungi, basidiomycetes attack and assist more intensely in disease reduction [19]. For example, [20] stated that with the use of phenolic compounds, hydrocinnamic derivatives had greater resistance towards Bayoud disease in the dead palm and some phenolic compounds can suppress *G. boninense* growth as well [20,21,22]. A recent study emphasized the physicochemical and anatomical changes occurring in the oil palm wood during *G. boninense* colonization, and suggested that treating oil palm stumps with benzoic acid could be a solution to reducing the *G. boninense* inoculum pressure during replantation in a sustainable manner [23]. Phenolic compounds are efficacious, naturally obtained fungicides, environmentally safe, with a few harmful residues and conservative for the natural ecosystem [24]. Therefore, this study was conducted to investigate the role of phenolic compounds on suppression, mycelial morphological alterations, membrane integrity and ergosterol estimation of *G. boninense*. The phenolic compounds selected to conduct this study were based on their ability to suppress *G. boninense* in preliminary experiments.

## 2. Materials and Methods

### 2.1. Microorganism, Culture Conditions and Treatments

The isolate *G. boninense* strain PER71 was obtained from the Malaysian Palm Oil Board (MPOB), Bandar Baru Bangi. The culture was maintained by sub-culturing it on the Potato Dextrose Agar (PDA, Oxoid-Chesire, UK) at regular intervals of 7 days and was incubated at 28 °C. Four phenolic compounds namely gallic acid (R&M Chemicals-London, UK), thymol (Bendosen, Norway ), carvacrol (Sigma-Aldrich, Burlington, MA, USA) and propolis (the crude (7 g) was dissolved in 80% ethanol) were obtained from Yi Wang Honey Garden (M) Sdn Bhd, Semenyih, Selangor and were utilized at different concentrations in this study.

### 2.2. Screening of Phenolic Compounds on Growth of G. boninense

To test the antifungal ability of each phenolic compound against *G. boninense* the range of concentrations was tested using the poison food technique. The concentrations of phenolic compounds were selected based on the preliminary study, to determine the mode of action. The concentrations were further narrowed down based on the screening results. The results collected from the poison food technique were subjected to probit analysis to identify the lethal concentration to justify the exact concentrations of each treatment based on the PIRG (%) values obtained from the calculation of LC_50_ and LC_90_ (POLO Plus-Probit and Logit Analysis-LeOra Software-version 0.03, Berkeley, CA, USA) and Microsoft Excel 365 (Washington DC, WA, USA).

#### Poison Food Technique

The phenolic compounds, namely GA, THY, CARV and PRO were added to the heat sterilized PDA (Oxoid) (39 g/L) media at 40 °C to obtain the required concentration. The concentration prepared for GA (5, 6, 7 and 8 mg/mL), THY (0.1, 0.15, 0.2 and 0.25 mg/mL), PRO (2, 2.5, 3 and 3.5 mg/mL) and CARV (0.1, 0.13 and 0.15 mg/mL) were then poured into 9 cm diameter Petri plates. The prepared plates were then allowed to solidify. A 5 mm mycelial agar plug was cut from actively growing seven-days old cultures of *G*. *boninense* and inoculated in the centre of each prepared plate. The plates only with PDA served as a control treatment. All of the plates were incubated at 28 °C until the control plate was completely colonized by *G*. *boninense*. The radial growth of the pathogen in all the plates was measured at two perpendicular points from the centre. The average values were recorded. The experiment was conducted in a quadruplicate. The percentage of inhibition of the radial growth was measured as per the equation below,

$$\mathrm{PIRG}\;\left(\%\right)=\frac{C-T}{C}\times 100$$

where,

C: radial growth of mycelia in the control plate, and
T: radial growth of mycelia in treatment plate.

### 2.3. Mechanism(s) Involved in the Suppression of G. boninense

#### 2.3.1. Electrolyte and Sugar Leakage

In a sterilized 150 mL conical flask, a 50 mL Potato Dextrose Broth (PDB) medium was added with respective phenolic compounds to get the desired concentrations. Each flask was then inoculated with a 5 mm mycelial plug excised from the 7-days old culture of *G. boninense* and sealed with parafilm. The flasks were incubated for 10 days at 25 ± 2 °C followed by filtering off the mycelium using Whatman No.1 filter paper (ϕ 6 mm). The collected mycelium was washed thoroughly with deionized water. The flask with only 50 mL PDB and without phenolic compounds served as a control.

##### Electrolyte Leakage Measurement

Each of the phenolic acid-treated mycelium (1 g) was added to a conical flask containing 40 mL of bi-distilled water. The flasks were then incubated in a shaking water bath at 25 °C for 8 h (Bathing solution). The conductance of the samples was recorded at 0 h, then at two-hour intervals with a conductivity meter (Eutech Conductivity Bridge, Eutech, COND 6+, ThermoFisher Scientific, Tokyo, Japan). One mL of chloroform was added at the end of incubation and total leakage of mycelium was recorded. Electrolyte leakage was expressed as μ mhosg^−1^ fresh weight of mycelium [25].

##### Sugar Leakage Measurement

The bathing solution that contained mycelia soaked for 8 h in the previous leakage analysis with different concentrations of phenolic compounds were processed according to the Anthrone-sulphuric acid method to determine the sugar leakages from the *G. boninense* mycelia. One ml of the bathing solution was combined with 2 mL of anthrone reagent [0.2 g anthrone, 8 mL absolute ethyl acetate, 20 mL distilled water and 100 mL H_2_ SO_4_ (density = 1.84) at 10–12 °C]. The mixture was then boiled for approximately 16 min, followed by the measurement of absorbance reading using a spectrophotometer (Thermo Scientific Multiskan Go, Tokyo, Japan) at 620 nm wavelength. The reaction mixture of the untreated control was used to set the absorbance to zero. The amount of reducing sugar was then calculated using a standard curve of authentic glucose. The sugar leakage was expressed as μ mhosg^−1^ fresh weight of mycelium. All treatments in the above experiment were replicated five times.

#### 2.3.2. Membrane Depolarization Assay

Three 5 mm plugs from a 7-days old culture of *G. boninense* were inoculated into a 150 mL conical flask containing 50 mL PDB with different concentrations of phenolic compounds. The inoculated flasks were incubated for 10 days at 25 ± 2 °C. PDB without any phenolic compound served as a control. The cultured broth was then centrifuged at 9500 rpm for 10 min to harvest the cells (mycelium). To detect cell membrane destruction caused by phenolic acids, the cells were re-suspended in 1 mL of Phosphate buffered-saline (PBS)-pH7.4 followed by the addition of 5 μg of bis-(1,3-dibutyl barbituric acid) trimethine oxonol (DiBAC_4_(3)), [Blue laser (488 nm), channel FL1 525/40BP FITC channel]. The samples were then analyzed via a flow cytometer using Beckman Coulter Flow Cytometer [26]. Results were analyzed using CytExpert Software version 2.4.0.28 (Brea, CA, USA)

#### 2.3.3. Membrane Permeability Assay

The fungal cell of *G. boninense* was prepared as described in the above section (Membrane depolarization assay) via 6 μm propidium iodide (PI) influx assay. The harvested cells were re-suspended in 1 mL of Phosphate buffered-saline (PBS)-pH7.4 followed by the addition of PI. Then, the samples were incubated for 5 min at room temperature. [Yellow/green laser (561 nm), channel FL3 585/42BP, PE channel] was used for PI influx assay. The uptake of the PI by the *G. boninense* fungal cells was analyzed using the Beckman Coulter Flow Cytometer to identify the fungal membrane permeability [26]. Results were analyzed using CytExpert Software.

#### 2.3.4. Ergosterol Determination

A 100 mL conical flask containing 50 mL Malt Extract Broth (MEB) was added with different concentrations of phenolic compounds. The flasks without any phenolic compounds served as the control medium. All the flasks were inoculated with 3 (5 mm mycelial plugs of *G. boninense*) from 7-days old cultures and sealed to avoid contamination. The inoculated flasks were kept at 25 ± 2 °C. After 2 weeks of the incubation period, the mycelia were removed from the flask and rinsed with sterile distilled water. Then, the mycelia were air-dried under the laminar airflow and stored at −80 °C before use.

Microwave-assisted extraction opted for ergosterol determination [27]. Briefly, the dried mycelium was macerated with a sufficient amount of liquid nitrogen to form a powder. The powders were then kept in the respective pyrex test tube with a Teflon screw cap added with 2 mL of methanol (chromatography grade) and 0.5 mL of 2 M sodium hydroxide and sealed tightly. The pyrex test tubes were placed in the conventional microwave (Dimension 4, National, Osaka, Japan), and subjected to microwave (Temp: 70 °C; Power: medium-high; Exposure time: 30 s). The mycelial solution was cooled and neutralized with concentrated hydrochloric acid using the pH meter (Eutech Instruments, Thermofisher Scientific). Following this, the solution was transferred to pear-shaped bottom flasks and was extracted 3× with 2 mL of pentane (analytical reagent grade). The solution was evaporated to dryness with Buchi Rotavapor R-200 with 834 vacuum pressure. The resultant crudes were dissolved in 500 μL of methanol. All the standard and treatment solutions were filtered using Whatman 0.45 μm nylon syringe filter before the HPLC analysis. The ergosterol standard was prepared by dissolving the ergosterol (Sigma-Aldrich, Burlington, MA, USA) with acetonitrile: methanol (50:50 *v*/*v*) solvent mixture with optimum temperature on the hot plate with the help of a magnetic stirrer. The concentrations of the standard ergosterol were adjusted to 10, 100 and 1000 ppm.

#### 2.3.5. Alteration in Structural Morphology

##### Scanning Electron Microscopy (SEM)

Scanning electron microscopy (SEM) was used to examine the effect of the phenolic compounds on the mycelial morphology of *G. boninense.* The SEM viewing samples were prepared by peeling the surface off each of the *G. boninense* cultures grown on PDA poisoned with different concentrations of each phenolic compound. Excised cultures of 1 cm (5 each) were immediately fixed with 4% glutaraldehyde for 12 to 24 h. The fixed cultures were then washed with 0.1 M sodium cacodylate buffer and fixed with osmium tetroxide for 2 h followed by dehydration for 10 min in a pure alcohol series (each 10 min, 1×) with different concentrations of 30%, 40%, 50%, 60%, 70%, 80%, 90% and 100% (15 min, 3×). After that, the prepared samples were dipped in 100% acetone three times (15 min each time). The samples were then critical-point dried with liquid CO_2_ and coated with gold-palladium. The coated mycelial cultures were viewed using SEM (JEOL, JSM 6400, Tokyo, Japan) operating at 15 kV.

##### High-Resolution Transmission Electron Microscopy (HR-TEM)

The surfaces peeled off mycelial samples were excised, using the method described in the section above, and were fixed with 6% glutaraldehyde for 24 h at room temperature. This was followed by rinsing three times with 0.02 M phosphate buffers and subsequently fixed with 2% osmium tetraoxide for 24 h at 20 °C, dehydrated in HPLC graded ethanol series for five minutes, CO_2_ dried (SAMDRI 780-B Tousimis, Rockville, MD, USA) and sputter-coated with gold-palladium in a Nanotech sputter coater (BAL-TEC SDC 050, New York City, NY, USA). Coated mycelial samples were kept in a desiccator until examination. The samples were embedded in resin (EMS cat.14380) and kept for polymerization. Then, Ultra-thin sections (60 nm thick) were cut with a diamond knife (Ultracut R, Leica, Washington, DC, USA) and collected in Formvar coated copper grids (EMS cat. FCF200). They were contrasted in uranyl acetate and lead citrate, washed and then examined via JEM-2100F Field Emission Electron Microscope [28].

#### 2.3.6. Statistical Analysis

Data analysis was conducted using SAS Statistical Software, version 9.4 English. A *p*-value of ≤0.05 was considered significant. The means were compared using Tukey’s Studentized Range (HSD) Test. The experiment was carried out at the probability level of 95% (α = 0.05). One-way Anova was carried out on the combinations of phenolic compounds and their concentrations. The results were expressed as the mean ± standard error obtained from the replicates.

## 3. Results

### 3.1. Screening of Phenolic Compounds on the Growth of G. boninense

#### 3.1.1. Poison Food Technique

The four phenolic compounds at different concentrations were tested for their significant inhibition of the mycelial growth of *G. boninense* (Table 1). Gallic acid at 8 mg/mL gave the best inhibition of *G. boninense* with a PIRG of 94% followed by THY with 87.13% inhibition at the concentration of 0.25 mg/mL. For PRO and CARV, the PIRG was 32.6%, and 36%, respectively, at their highest concentration tested as compared to the growth in the control plates. Generally, GA and THY were the most effective phenolic compounds that can control the *G. boninense* growth compared with other phenolic compounds tested. There were significant differences (*p* < 0.05) between the phenolic compounds tested. The higher concentrations of each phenolic compound exhibited a higher antifungal effect. The inhibition percentage showed a high deviation due to the various concentration used to treat *G. boninense*.

##### Lethal Concentration (LC_50_ and LC_90_)

The comparison between tested phenolic compounds was further confirmed by comparing their minimum inhibitory concentrations, LC_50_ and LC_90_ values (Table 2). Gallic acid exhibited inhibition at 5.25 mg/mL (LC_50_), and 8.51 mg/mL (LC_90_). The highest inhibitory effect observed in Table 1 corresponded with GA in probit analysis in Table 2. Across the probit analysis, the minimum concentration required for the inhibitory effect of target fungi, *G. boninense* is at a higher range for PRO and CARV compared to GA and THY. The r-value ranged between 0.86 and 0.99, indicating a strong correlation between the phenolic compounds and mycelial growth inhibition. The R square values showed substantial values and some variation in concentrations effecting the growth of *G. boninense*.

### 3.2. Mechanism(s) Involved in Suppression of G. boninense

#### 3.2.1. Electrolyte and Sugar Leakage

Table 3 indicates the electrolyte leakage from the mycelia treated with phenolic compounds. All the phenolic compounds had a significant ‘*p* < 0.05’ effect on the mycelial membrane of the *G. boninense*. Electrolyte leakage in GA-treated mycelium was consistently higher at all tested concentrations. The maximum leakage was 100.30 μ mhosg^−1^ at the concentration of 8 mg/mL of GA-treated *G. boninense.* GA-treated mycelium showed significantly higher electrolyte leakage compared to other phenolic compounds. The fungal mycelium treatment with THY has resulted in achieving leakage value (61.30 μ mhosg^−1^), whereas, PRO and CARV caused the least amount of leakage at all the tested concentrations.

The sugar leakage from the *G. boninense* mycelia treated with phenolic compounds is indicated in Table 4. All the phenolic compounds had a significant effect on the membrane of the *G. boninense* with different efficacies. Gallic acid at 8 mg/mL recorded a high amount of sugar leakage (0.985 μ mhosg^−1^) as compared to the control (0.0324 μ mhosg^−1^). The ascending order of the efficiency of the phenolic compounds in affecting the fungal membrane and sugar leakage is recorded as CARV, THY, PRO and GA.

#### 3.2.2. Membrane Depolarization Assay

**Figure 1:**
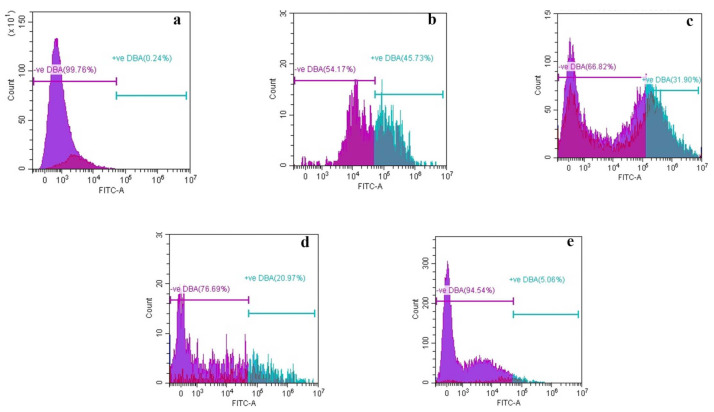
Histogram of cell viability determined by flow cytometry in (**a**) control; (**b**) Gallic acid; (**c**) Thymol; (**d**) Propolis and (**e**) Carvacrol treated cells (mycelium) at the highest tested concentration. The violet region represents the live cells and the blue region refers to dead cells. The fluorescence of 10,000 events of membrane potential was measured by a flow cytometer.

It is deduced that 0.24% of *G. boninense* cell mortality was observed in the control. This could be due to the presence of some debris in the sample (Figure 1a). In contrast, GA-treated cells exhibited 45.73% of mortality, which is the highest among all the phenolic compounds (Figure 1b). Similarly, 31.90% cell mortality was observed in THY (Figure 1c). GA and THY significantly inhibited the growth of the pathogen, resulting in a lower number of total cells available in the sample. Moreover, 20.97% and 5.06% of dead cells were recorded with a high percentage of live fungal cells in PRO and CARV treatment, respectively (Figure 1c,d).

#### 3.2.3. Membrane Permeability Assay

**Figure 2:**
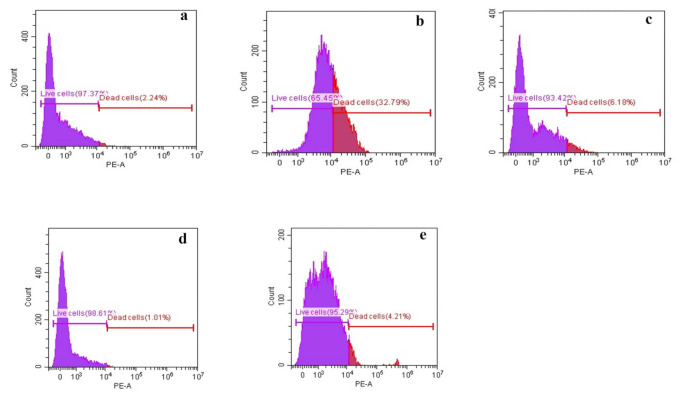
Histogram of cell viability determined by flow cytometry in (**a**) Control; (**b**) Gallic acid; (**c**) Thymol; (**d**) Propolis and (**e**) Carvacrol treated cells (mycelium) at the highest tested concentration. The blue region represents the live cells and the light blue region refers to dead cells. The fluorescence of 10,000 events of membrane potential was measured by the addition of PI via flow cytometer.

The disruption of cell membrane permeability is depicted in Figure 2. Cell membrane permeability of the fungal mycelium was assessed as the fluorescence intensity due to the reaction of PI. The utilization of PI into the DNA proves that the fungal cell nucleus has been stained with PI due to severe damage and the formation of pores in the cell membrane. Cells excised from the control exerted about 2.24% of dead cells, which is considered as debris at the 0-axis, accounted as negligible. Cells treated with GA, THY, PRO and CARV exerted 33%, 6.18%, 1.01% and 4.21% dead cells, respectively (Figure 2). The cells treated with phenolic compounds resulted in a higher % of dead cells, accumulation of compounds, thus having high levels of PI intake, whereas, in the control, the % of dead cells is lower than 5%. The fungal cells treated with GA exhibited the highest % of mortality and more nucleus staining was observed compared to other treatments (Figure 2b). THY treated cells exerted less staining of the nucleus (Figure 2c). Figure 2d,e represents the cells treated with PRO and CARV respectively and exhibited less than 5% of PI intake and showed less damage occurred in the cell membrane. These results proved that GA treated *G. boninense* was significantly prominent in cell death compared to control and other phenolic compounds. The percentage of mortality could also be varied due to membrane porosity and potassium ions released from the membrane.

#### 3.2.4. Ergosterol Determination

The amount of ergosterol influenced by phenolic compounds was determined using high-performance liquid chromatography. The amount of ergosterol extracted from the control was 122.26 ppm. The GA-treated *G. boninense* exerted 96.77 ppm of ergosterol. Similarly, 162.76 ppm, 158.09 ppm and 382.30 ppm ergosterol were detected in THY, PRO and CARV respectively. The ergosterol concentrations were correlated with the peaks, whereby it gave a good correlation coefficient (r) of 0.9984 showing a strong positive correlation. The peak areas were examined for linearity within the level of 10–100 ppm ergosterol standard. The amount of ergosterol for the mycelium treated with and without phenolic compounds is included in Table 5. The retention time for the ergosterol detected in control was 8.26 min.

#### 3.2.5. Alteration in Structural Morphology

##### Scanning Electron Microscopy (SEM)

**Figure 3:**
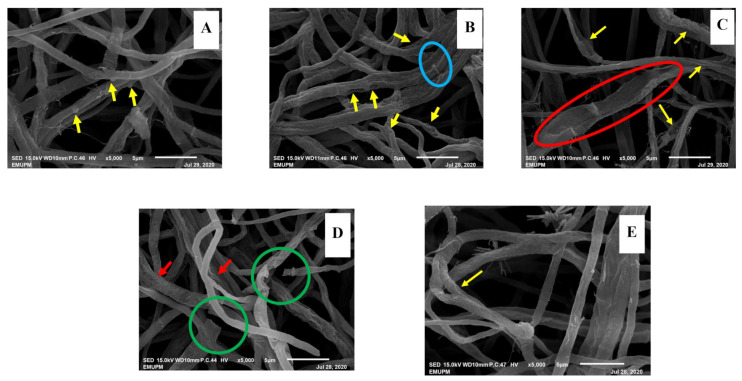
The alterations in mycelial morphology after treatment with phenolic compounds were observed under 5000× magnification. (**A**) Healthy mycelium of *G. Boninense* excised from control plate. The arrows indicate the clamp connection; (**B**) Mycelium of *G. boninense* treated with gallic acid. The arrows indicate the fused mycelium with holes and shrinkage of the mycelium and the hyphae is encircled. (**C**) Mycelium of *G. boninense* treated with thymol. The arrows indicate the holes, shrivelling, empty hyphae and the encircled mycelial region indicates the rupturing of hyphae; (**D**) Mycelium of *G. boninense* treated with propolis. The arrows indicate the shrivelling, collapse and the encircled region indicated the breaking/detachment of mycelium connection. (**E**) Mycelium of *G. boninense* treated with carvacrol showing shrivelling of the mycelium (arrow).

**Figure 4:**
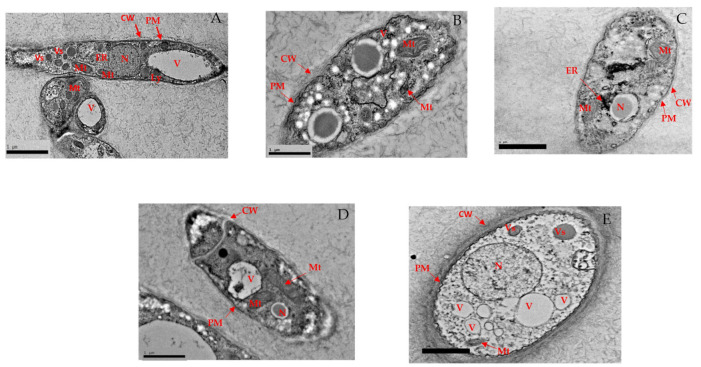
Ultrastructural modifications in hyphae of *G. boninense* treated with phenolic compounds observed under HR-TEM. (**A**) rigid and intact cell wall and plasma membrane, prominent cytoplasmic organelles, such as mitochondria, vesicles, vacuole and endoplasmic reticula as well as the nucleus in healthy hyphae. (**B**) The arrows indicate disruption of the cell wall and membrane, loss of vesicle with empty regions, condensed nucleus, deformed mitochondria and disorganization of cytoplasmic contents in hyphae treated with 8 mg/mL GA; (**C**) indicates the cell component leakage, disruption of the cell membrane, disorganization of cytoplasmic content in hyphae treated with 0.25 mg/mL THY; (**D**) shows the disruption of the cell membrane, disorganization of cytoplasmic content and less disintegration of the cell wall and plasma membrane; cytoplasmic organelles evident in hyphae treated with 3.5 mg/mL of PRO; and (**E**) less damage to fungal hyphae with intact cell wall and irregular but continuous plasma membrane, prominent nucleus and cytoplasmic organelles in hypha treated with 0.15 mg/mL of CARV. CW: cell wall, PM: plasma membrane, Mt: Mitochondria, ER: Endoplasmic reticulum, V: vacuole, N: Nucleus, Vs: Vesicle.

Scanning electron microscopic examination of the mycelium grown in the media without phenolic compounds exhibited smooth, dense, healthy mycelium with 1.244 μm diameter, and clear clamp connections (Figure 3A). The mycelium in GA treatment was less dense, shrivelled and fused (2.732 μm diameter), holes on the wrinkled surface of the hyphae were prominent (Figure 3B). Thymol treated mycelium was distorted (2.005 μm diameter), hyphae dominated by fused holes exposing the empty hyphae (Figure 3C), PRO treatment caused shrinkage (1.528 μm, diameter), the collapse of hyphae and detachment of mycelium (Figure 3D), whereas, CARV treatment did not cause much damage to the mycelial structure, only shrivelled mycelium(0.709 μm diameter) were observed (Figure 4E).

##### High-Resolution Transmission Electron Microscopy (HR-TEM)

Ultrastructural modifications of the cell wall and cell interior of phenolic compounds-treated hyphae were revealed by HR-TEM. The mycelium excised from the media without phenolic compounds exhibited rigid and intact cellular membrane, clearly representing cytoplasmic organelles, such as mitochondria and endoplasmic reticula as well as the nucleus. The cell membrane appeared as a sharp, electron-dense lipid bilayer closely attached to the inner face of the fungal cell wall (Figure 4A). Major changes due to phenolic compounds treatment were the accumulation of electron-dense inclusions in the cell wall and inner space between the cell membrane and the cell wall. The membrane structure of the mycelium treated with GA had an irregular shape, disruption in the cellular membrane, with fused and ruptured vacuoles. Many electron-dense inclusions, varying in size and shape were observed (Figure 4B). The hyphae treated with THY (Figure 4C) resulted in severe damage to the cell wall and cytoplasmic organelles. Most of the parts of cell wall and cell membrane of the cell were lost (or hardly observed). Similarly, PRO (Figure 4D) shows the disintegration of the organelles, damaged, blurry walls and detached plasma membrane from the cell wall structure. Hyphae treated with CARV showed a lesser amount of content in the nucleus and certain regions of cell membranes were disrupted with irregular margins, displayed blurry walls and damaged plasma membrane (Figure 4E). In addition to these rather drastic morphological or ultrastructural changes, impairment of membrane structures in the cytoplasmic organelles and nucleus were occasionally observed.

## 4. Discussion

Currently, the BSR disease is severely infesting the oil palm in all the palm growing countries and various methods are being considered and carried out to control the disease. However, none of the efforts can completely suppress and eradicate the infection caused by the *G. boninense*. This study was conducted to identify potential phenolic compounds to suppress the growth, of *G. boninense* and identify the main mechanism involved in the suppression of the pathogen.

The observation made from PIRG (%) revealed the suppressive effect of phenolic compounds on *G. boninense*. Gallic acid treatment led to the highest inhibition at the maximum tested concentration. Gallic acid could exhibit both pro-oxidant as well as antioxidant characteristics, and its pro-oxidant action is responsible for its potent antifungal effect. The incorporation of GA might have auto oxidated to produce significant levels of H_2_O_2_ and O_2_ in the fungal mycelium. These increased reactive oxygen species (ROS) levels could cause mitochondrial potential loss, presence of intracellular Ca^2+^ leading to the disruption of the fungal growth, damage in the cellular components exchange from the nutrition source and also an unconducive environment causing cell death [29]. Similarly, THY at the highest tested concentration showed significant inhibition of *G. boninense* growth. It also caused severe damage to the cell wall of the fungal mycelium by rupturing the cell wall and accumulating cellular components, as evident in this study.

Furthermore, the presence of phenolic compounds caused insufficient nutrient exchange for the cell wall outer membrane-bound organelles and caused damage to the thin outer membrane. Nuclear disruption and enlargement could be due to the elevation of oxygen consumption owing to the accumulation of ROS [30]. Not only that, the higher concentration may be toxic to the pathogenic fungi and suppress its growth [31]. The diffusible compounds in the PRO treatments played some role in suppressing *G. boninense* growth. For example, the presence of the resin obtained after the extraction of propolis had an antifungal effect on *G. boninense*. Resin is a compound obtained naturally from propolis, is known to have antifungal properties [32]. Carvacrol is associated with hydrophilic properties with the presence of the OH group and enhances the antifungal properties. Nevertheless, THY, which has a carvacrol isomer and the p-cymene derivative found in thymol, exerts antimicrobial efficiency towards pathogens [33]. However, the resin in PRO and CARV does not prove to be detrimental to *G. boninense* in the present finding.

The comparison between tested phenolic compounds was further confirmed by comparing their MIC values against *G. boninense* growth. The LC_50_ and LC_90_ values for THY and CARV were low. In GA, it was a little low too and it could be due to its molecular structures. It is known as a triphenolic compound with low molecular weight and due to the presence of the hydroxyl group, it dissolves in water and is bioavailable [34]. Similarly, thymol and carvacrol both consist of isopropyl groups, hydroxyl groups and delocalized electrons that can easily dissolve and accumulate in the cell membrane causing destabilization in the membrane [24]. In propolis, various classes of chemical compositions were reported by Toreti and colleagues in 2013 and are known to possess antimicrobial activity towards targeted pathogens, in particular, the pinocembrin compound was found to be the best. Therefore, various ranges of concentrations are required to execute antifungal properties due to different components and chemical compositions such as phenolic constituents, terpenoids and flavonoids found in PRO [35]. The LC_50_ and LC_90_ range of propolis and carvacrol were very high due to the low concentration to fight with the 50% of the *G. boninense* population. Additionally, studies showed that a lower concentration of propolis was used to test the antifungal properties. As this experiment was used as a preliminary study, several ranges of concentrations were chosen to identify the antifungal efficacy of propolis and carvacrol to inhibit the growth of *G. boninense*. Furthermore, this shows that a higher concentration is required for certain phenolic compounds to exert their efficacy in suppressing the *G. boninense*.

The antimicrobial activity of plant-derived phenols has been partly attributed to their hydrophobicity, rendering the membrane permeability and leakage of cell contents. The interaction between phenolic compounds and the fungal membrane has facilitated the penetration of intracellular components through the membrane and the formation of pores. Furthermore, enhancement of phospholipid asymmetry loss due to GA could have caused the detachment of plasma membrane together with translocation of phosphatidylserine (PS) [30]. Thus, cytoplasmic membrane collapse and leakage of cellular contents eventually lead to cell death [36]. According to [30] altered plasma membrane due to the formation of aqueous pores could also lead to cell death. PI and DBA staining suggested that all the tested phenolic compounds could induce cell membrane damage in the *Ganoderma* cells, which further resulted in electrolyte leakage from cells. The GA (Table 3 and Table 4) exerted a higher amount of leakage for both electrolyte and sugar due to severe damage of the membrane as shown in Figure 3B and Figure 4B followed by THY, which acted similarly. Our findings are in corroboration with [37] who reported that the GA-treated fungal cell, *Candida albicans* has shown loss of full form of fungal cell and damage on the cytoplasm. It has been reported that fungal cells treated with THY at 64–500 mg·mL^−1^ could be stained extensively by PI as compared to the control. In addition, researchers found that THY at 750 μg·mL^−1^ could induce the disruption of the cell membrane in *Salmonella ser. typhimurium*. The SEM and HRTEM findings further validate this and are in corroboration with those reported, significant leakages of cellular materials and altered morphology of bacterial cells induced by THY [38]. Similar findings proved that phenolic compounds affect the electrostatics state of the cell membrane and membrane integrity. The mycelial features were similar corresponding to [39].

The phenolic compounds specifically GA and THY had the greatest effectiveness in destroying the fungal morphology internally and externally. The integrity of the cell membrane was carried out via flow cytometer analysis and high-performance liquid chromatography to determine the PI influx, membrane depolarization and ergosterol content. The effect of phenolic compounds on morphological alterations correlated well with membrane integrity [40]. In comparison to all the phenolic compounds used in this study, GA caused significant damage to the morphology and structural integrity followed by THY, PRO and CARV. These phenolic compounds had a tremendous effect on the *G. boninense* cell membrane by exerting holes, damaging the cell size, and its capacity to carry out the biological pathways. GA and PRO affected the mycelial normal growth and affected the ergosterol amount in the fungal cell. GA and THY affected the permeation of (PI), particularly following short incubation periods, indicating the mechanism of action of the phenolic compounds as a primary lesion on the cell membrane, leading to cell death. Hence, it can be concluded that the observed fungicidal activity of GA and THY, on the mycelium was due to severe lesions on the membrane, resulting from direct damage to the cell membrane. The higher number of dead cells in GA and THY treated cells could be due to the splitting of the cells, which are dispersed as smaller cells, thus, exhibiting higher mortality. Overall, a study suggested that H_2_O_2_ stress induction could increase the germination and extension of hyphae and be beneficial for cell wall synthesis despite testing in lower concentrations of phenolic compounds to understand a better antimicrobial pattern [41,42] as GA has more antifungal activity towards *G. boninense* and leading to less number of viable cells. As for the PRO and CARV, the efficacy towards the *G. boninense* was not too intense, thus, membrane integrity and permeability have less significance in comparison to both. In the sense of membrane depolarization and permeability assay, GA was very effective compared to other phenols. It is understood that GA had a strong antifungal effect causing less mycelial growth, whereas THY, CARV and PRO exhibited more mycelia, therefore the amount of dead and live cells varied due to its population in this case study.

Ergosterol is the major fungal membrane sterol that regulates membrane fluidity, biogenesis of plasma membrane, thickness, permeability and biosynthesis of ergosterol [43]. The purpose of the sterol helps in the fungal membrane structural integrity and to maintain the cell’s rigidity. Therefore, any damage occurred to the cell membrane, interferes with the natural and usual routine of the cell and interrupts the internal components in it [44]. The production of ergosterol is usually initiated by the presence of CYP51 enzyme, whereas reduced production of it led to disruption of ergosterol. Thus, this leads to the antifungal mechanism, whereby, inhibition of the CYP51 enzyme inhibits the fungal membrane ergosterol [45]. The comparison of the retention time and the absorption spectrum of the standard compound identified the antifungal mechanism. The ergosterol content and membrane permeability and integrity interrelate with each other, whereby, alterations in cell wall and membrane will disrupt the interchange of hydrophobic and hydrophilic components, thus, proving the effectiveness of phenolic compounds. Very significant inhibition of ergosterol biosynthesis was observed in the GA-treated mycelium, implying that this fungus is tolerant to the inhibition of ergosterol biosynthesis and has an effect on membrane integrity. For example, as stated by [37], GA can bind to the membrane ergosterol and causes a decrease in the ergosterol amount. It was reported that GA obtained naturally can cause a decreased amount of ergosterol due to strong intermolecular H-bonding and also unpaired electron spin density that is delocalized [37,38]. In fungi, GA inhibits the ergosterol biosynthesis and reduce the activity of sterol 14α-demethylase P450 (CYP51) and squalene epoxidase while for cell leakage the interference would be towards the β-1,3-glucan synthase thus unable to hold the internal organelles, leach them out and affect the cell growth [39,43]. Sterol and phospholipid interaction in cytoplasmic membrane affect the fluidity and disrupt the transport of membrane materials across the fungal membrane. This may result in a decrease in the uptake of azoles from phenolic compounds and change the sterol and phospholipid composition. The scenario is similar to GA treatment in ergosterol extraction as the amount is lower compared to control, whereby the accumulation of azoles in the intracellular region can be reduced by the lack of penetration of phenolic compounds due to a low amount of ergosterol in the plasma membrane, which may have defects in the membrane barrier [46].

Moreover, the ergosterol content was higher in the mycelium treated with the rest of the phenolic compounds. This could be due to the disruption of ergosterol biosynthesis at different stages, accumulation of ergosterol and induction of transcriptional responses [47]. In conjunction with [48], it was deduced that disruption at sterol intermediates leads to stress responses towards ergosterol biosynthesis responses. The degree of antifungal activity is relatively influenced by the position of the hydroxyl group on the phenolic ring [38]. It could be inferred that the cellular damage is not always directly towards biosynthesis of ergosterol but in any different steps of the biosynthetic pathway and other sterols present in the fungal membrane that affect the transcription and stress responses. Therefore, it is suggested that GA and THY act at the level of the fungal membrane, increases membrane permeability and cause the depletion of components essential to fungal survival [49,50]. This results in an irregular amount of ergosterol due to accumulation, disruption and unusual leaking according to the fungal membrane pore affinity with the phenolic compounds [37].

Structural alterations observed via SEM and HRTEM revealed that the phenolic compounds play an important role in altering the structural morphology, the outer surface, internal organelles and cytoplasmic components of the mycelium. Among all the compounds, GA and THY affected the mycelium severely by imposing holes, shrinking the mycelium structure, shrivelling the outer membrane, and severely disrupting the cellular membrane, cell wall and damage the internal cytoplasmic components such as the nucleus, mitochondria, endoplasmic reticulum, vacuoles and vesicle. These majorly describe the severity of the chemical pathways and biological activities of the mycelium [51]. Gallic acid and THY severely affected the mycelium compared to PRO and CARV. Gallic acid and THY targeted the cell membrane and disrupted cell wall integrity [52]. The cell wall is necessary for cell division but in this study, due to severe disturbance by the phenolic compounds, the mycelium lost the integrity of the cell membrane, thus was unable to survive the normal environmental condition to regulate the synthesis of biological activities and enzymes [52,53]. It has been suggested that propolis-detachment to the fungal cell wall, disturbance of division was due to interaction with cellular sulphydryl compounds. Moreover, [54] discussed the disruption in germ tube pathways and inhibition of yeast growth due to the phenolic compounds. They further explained that invagination of the plasmalemma disrupts the dynamic relationship between ergosterol and chitin biosynthesis due to phenolic compound treatment. The mechanism of the antifungal effect depends predominantly on the ability to affect the function of cellular lipoprotein membranes, causing impairment of cellular ionic homeostasis, acidification of vacuolar and cytosolic pH, and even the destruction of structural cellular integrity [43,53]. A probable mode of alteration could be in the cell permeability, which explains the changes in the morphology and size of the internal organelles such as mitochondria and vacuoles [39] as found in current studies.

Phenolic compounds could interrupt the membrane potential for the exchange of internal and external components in and out of the cell [36]. Hydroxyl, amides and sulphonic groups in the phenolic compounds were suggested to have the ability to interact with the cell membranes [54]. Moreover, GA and THY could be promising drugs after improved formulations and also advocate the determination of optimal concentrations for industrial applications, as an alternative to synthetic fungicide for the treatment and prevention of basal stem rot disease.

## 5. Conclusions

Phenolic compounds are the potential determinant to inhibit the growth of the *G. boninense* as they alter the morphological characteristics of the fungus. In all the tested phenolic compounds, GA followed by THY were the best inhibitors of *G. boninense* and had an extreme impact on the cellular structure. This proved that suppression of *G. boninense* was due to the chemical nature of GA and THY, which worked best at their specific concentration. It is hypothesized that these lipophilic compounds penetrate the cell, disrupting the plasma membrane integrity, increase ion permeability and target the ergosterol biosynthesis pathway, thus, impairing its biosynthesis, which may result in cell death.

These phenolic compounds could be proficient in minimizing the stress caused by basal stem rot disease to oil palm industry, in a environmentally friendly manner. Further nursery and field evaluation are required to validate their consistency in BSR disease suppression. Moreover, identification of toxicity levels of the phenolic compound in relation to the oil palm should be tested to sustain the growth of the palms and minimize the hazardous impact on the environment if any.

## Figures and Tables

**Table 1:** Percentage Inhibition of Radial Growth of *Ganoderma boninense* as influenced by various phenolic compounds using poison food technique.

Treatments (mg/mL)	PIRG (%)
Control	0 ^e^
GA 5	50.14 ± 3.8 ^c^
GA 6	63.08 ± 3.8 ^b^
GA 7	62.5 ± 3.8 ^b^
GA 8	94 ± 3.8 ^a^
THY 0.1	9.09 ± 3.76 ^e^
THY 0.15	55.96 ± 3.76 ^c^
THY 0.2	74.86 ± 3.76 ^b^
THY 0.25	87.13 ± 3.76 ^a^
PRO 2	35.88 ± 3.76 ^d^
PRO 2.5	34.23 ± 3.76 ^d^
PRO 3	32.6 ± 3.76 ^d^
CARV 0.1	31.62 ± 3.76 ^d^
CARV 0.13	33.2 ± 3.76 ^d^
CARV 0.15	36 ± 3.76 ^d^

Means with different letters within the same column are significantly different between treatments (*p* < 0.05) and according to Tukey’s Studentized Range (HSD) Test. Each value represents a mean of five replicates ± indicates Standard error. GA = Gallic Acid, THY= Thymol, PRO = Propolis and CARV = Carvacrol.

**Table 2:** The Lethal Concentration (LC_50_ and LC_90_) of the phenolic compounds tested against *G. boninense*.

Phenolic Compounds	Lethal Concentration (mg/mL)			
	LC50	LC90	Correlation (r)	R Square
GA	5.25	8.51	0.86	0.74
THY	0.19	0.22	0.91	0.84
PRO	74.13	91.2	0.99	0.99
CARV	0.68	9.33	0.91	0.83

Each value represents a mean of four replicates. GA = Gallic Acid, THY= Thymol, PRO = Propolis and CARV = Carvacrol.

**Table 3:** Electrolyte leakage from the *G. boninense* mycelium under the influence of phenolic compounds.

Treatments (mg/mL)	Electrolyte Leakage in *G. boninense* Mycelium
Control	23.83 ± 3.7 ^f^
GA-5	79.73 ± 3.82 ^ab^
GA-6	79.33 ± 3.82 ^ab^
GA-7	92.43 ± 3.82 ^a^
GA-8	100.30 ± 3.82 ^a^
THY-0.1	42.10 ± 3.72 ^d^
THY-0.15	37.77 ± 3.72 ^e^
THY-0.2	52.27 ± 3.72 ^c^
THY-0.25	61.30 ± 3.72 ^b^
PRO-2	43.43 ± 3.72 ^d^
PRO- 2.5	44.8 ± 3.72 ^d^
PRO-3	31.70 ± 3.72 ^e^
PRO-3.5	30.27 ± 3.72 ^e^
CARV-0.1	32.47 ± 3.72 ^e^
CARV-0.13	22.8 ± 3.72 ^f^
CARV-0.15	23.53 ± 3.72 ^f^

Means with the same letter are not significantly different within the column. Each value represents a mean of four replicates. ± indicates Standard error. GA = Gallic Acid, THY= Thymol, PRO = Propolis and CARV = Carvacrol.

**Table 4:** Sugar leakage from the *G. boninense* mycelium under the influence of phenolic compounds.

Treatments (mg/mL)	Sugar Leakage of *G. boninense* Mycelium
Control	0.0324 ± 0.32 ^c^
GA-5	0.1654 ± 0.33 ^b^
GA-6	0.0345 ± 0.33 ^cd^
GA-7	0.1385 ± 0.33 ^b^
GA-8	0.985 ± 0.33 ^a^
THY-0.1	0.0923 ± 0.32 ^b^
THY-0.15	0.1288 ± 0.32 ^b^
THY-0.2	0.0567 ± 0.32 ^c^
THY-0.25	0.0834 ± 0.32 ^b^
PRO-2	0.0569 ± 0.32 ^c^
PRO- 2.5	0.0256 ± 0.32 ^cd^
PRO-3	0.0988 ± 0.32 ^b^
PRO-3.5	0.2973 ± 0.32 ^b^
CARV-0.1	0.0188 ± 0.32 ^d^
CARV-0.13	0.0963 ± 0.32 ^b^
CARV-0.15	0.0945 ± 0.32 ^b^

Means with the same letter are not significantly different within the column. Each value represents a mean of four replicates ± indicates Standard error.

**Table 5:** The table shows the amount of ergosterol extracted from the *G. boninense* treated with phenolic compounds.

Phenolic Compounds	Retention Time (min) Ergosterol	Amount (ppm) Ergosterol
Control	8.337	122.26
GA	8.200	96.77
THY	8.173	162.76
PRO	8.253	158.09
CARV	8.173	382.30

GA = Gallic Acid, THY= Thymol, PRO = Propolis and CARV = Carvacrol.

## Data Availability

Not applicable.
